# Marijuana as a Cause of Diffuse Coronary Vasospasm Leading to Cardiac Arrest

**DOI:** 10.7759/cureus.38026

**Published:** 2023-04-23

**Authors:** Muhammad Atif Khan, Faiza Humayun Khan, Hina Benish Khan, David Brabham

**Affiliations:** 1 Internal Medicine, University of Kansas Medical Center, Kansas City, USA; 2 Internal Medicine, Khyber Medical University, Peshawar, PAK; 3 Physiology, Khyber Medical College, Peshawar, PAK; 4 Internal Medicine, Texas Tech University Health Sciences Center, Amarillo, USA

**Keywords:** st myocardial infarction, marijuana use, illicit drugs, coronary spasm, cardiac arrest

## Abstract

Marijuana is considered as the most popular illicit drug around the world. It has numerous cardiovascular effects with myocardial infarction (MI) being a lethal one. The negative physiological effects of marijuana are well-studied, including tachycardia, nausea, memory impairment, anxiety, panic, and arrhythmia. We present a case of cardiac arrest following marijuana use in a patient who had a normal electrocardiogram (EKG) on presentation but diffuse coronary vasospasm on left heart catheterization (LHC) with no obstructive lesion. The patient had a transient episode of ST elevation on EKG following the procedure which resolved with an increased dose of nitroglycerine drip. Synthetic cannabinoids are more potent and not detected on a regular urine drug screen (UDS). In patients with low risk for cardiovascular events, particularly young adults, presenting with symptoms of MI/cardiac arrest, marijuana-induced MI should be suspected due to the severe adverse effects of its synthetic component.

## Introduction

Marijuana has been the most popular illicit substance in the United States (US) and the rest of the world. The National Survey on Drug Use and Health (NSDUH) reported that 18% of the US population above the age of 11 in 2020 has tried it once. The adverse effects of marijuana are well-known, including tachycardia, nausea, memory impairment, anxiety, panic attack, and arrhythmia. Myocardial infarction (MI) is a particularly lethal sequela of marijuana use. While marijuana lowers the anginal threshold in patients with preexisting heart disease, it may also do so to a sufficient degree to provoke MI in patients with no preexisting heart disease. Synthetic cannabinoids such as K2 and Spice are a rapidly emerging class of substances, usually undetectable on conventional urine drug screens (UDS), and becoming more widely available [1,2]. We present a case of cardiac arrest with diffuse coronary vasospasm and normal electrocardiogram (EKG) on presentation due to marijuana use. This case was previously presented as an abstract at the 2022 Southern Regional Meeting in New Orleans on February 10, 2022.

## Case presentation

**Figure 1:**
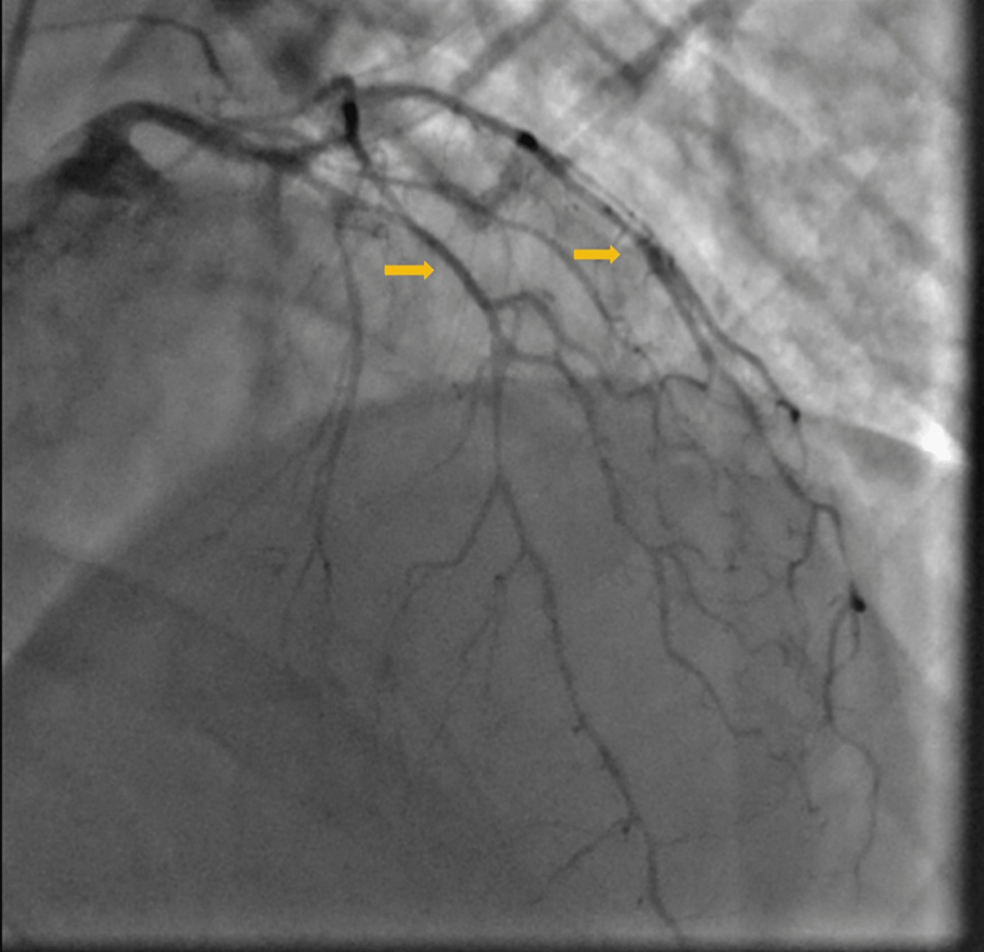
Coronary vessels diffuse spasms before intracoronary nitroglycerin (arrows pointing to multiple vessels to show diffuse nature)

**Figure 2:**
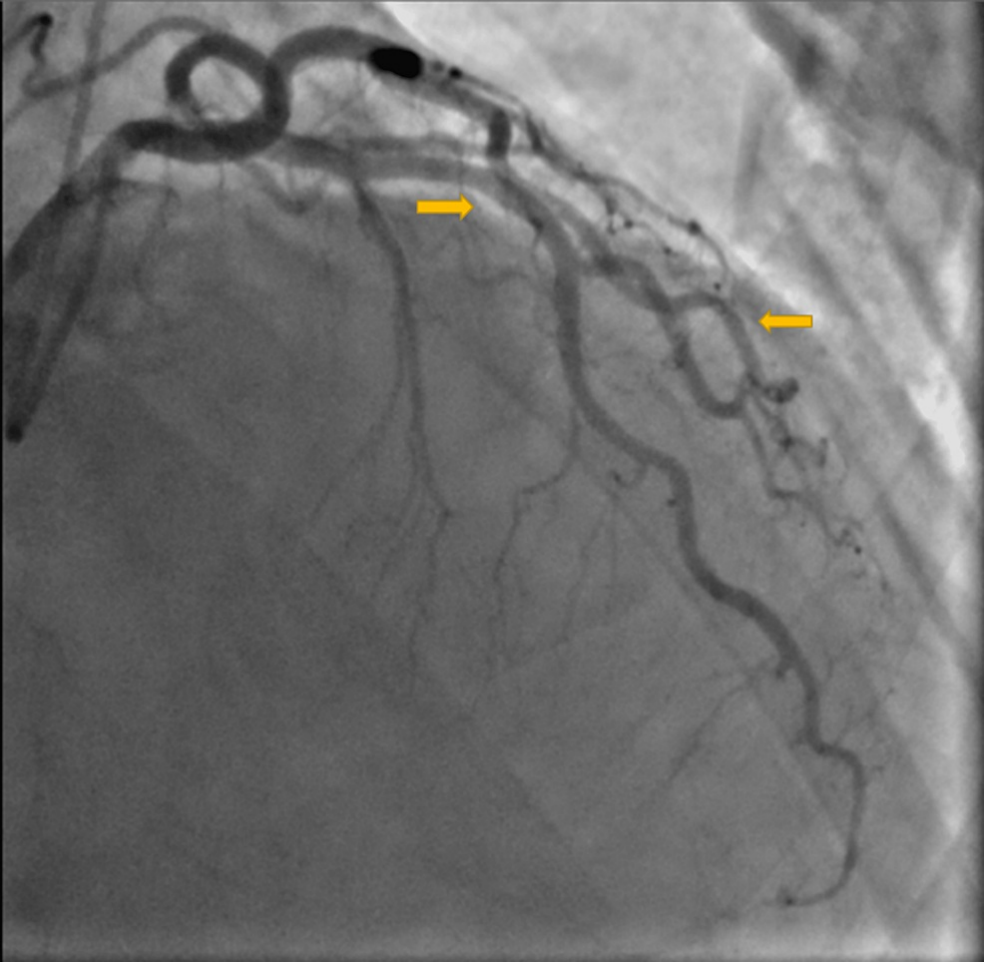
Coronary vessels dilatation after intracoronary nitroglycerin (arrows pointing to multiple vessels to show diffuse nature)

A 53-year-old male with a past medical history of hypertension, hyperlipidemia, and chronic obstructive pulmonary disease (COPD) presented with out-of-hospital cardiac arrest. The patient woke up with severe shortness of breath unresponsive to albuterol inhalers. Two hours later, the patient reported severe chest pain and was found pulseless. Emergency medical services (EMS) arrived and the patient was found to be in ventricular fibrillation. Cardiopulmonary resuscitation (CPR) was initiated, and the patient was intubated. The patient was shocked a total of five times, with four rounds of epinephrine and one round of amiodarone. Return of spontaneous circulation was achieved after 30 minutes. At the hospital, the patient’s blood pressure was 136/95 mmHg with no pressor support. Labs were significant for creatinine 1.5 mg/dl, bicarbonate 13 mmol/L, and lactic acidosis with pH 7.04. Troponins and brain natriuretic peptide(BNP) were within the normal range. EKG showed no dynamic ST segment changes on admission. UDS was positive for marijuana. The patient was urgently taken to the cath lab due to his presenting complaint of chest pain and ventricular fibrillation. Left Heart cath showed severe spasms involving all three coronary vessels responsive to intra-coronary nitroglycerin, but otherwise no significant obstructive coronary disease (Figures 1,2). He was started on a nitroglycerine drip for vasospasm.

**Figure 3:**
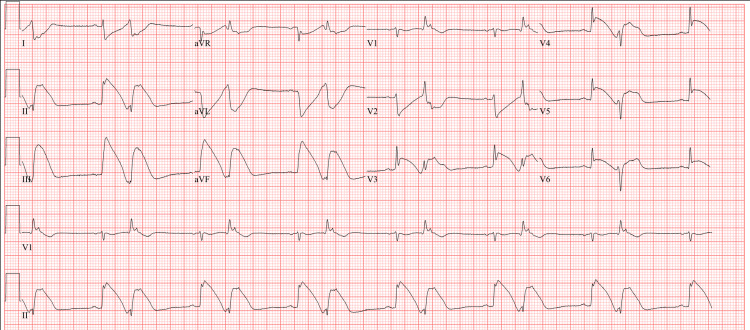
Transient changes on EKG following LHC EKG: Electrocardiogram; LHC: Left heart catheterization

Three hours later, the patient was found to have ST elevation in leads V3-V6 and wide QRS rhythm lasting for 20 minutes along with hypotension, 96/64 mmHg (Figure 3). At this point, the patient was intubated with good oxygen saturation and stable electrolytes. He was not on pressor support. There was no wheezing on physical exam. EKG changes resolved with increasing the rate of nitroglycerin drip and hypotension responded to IV fluids. Troponin high sensitivity (HS) drawn at the time was found to be elevated at 197 ng/L as compared to normal on admission. Repeat left heart catheterization (LHC) was not deemed necessary. Echocardiogram estimated the left ventricular ejection fraction at 45-50% with no wall motion abnormality. There were no further notable events, and the patient was discharged eight days after admission on aspirin, statin, and diltiazem with close follow-up with cardiology. He was counseled to quit marijuana. Due to no other plausible etiology, the diagnosis of marijuana causing severe global coronary vasospasm was entertained with the possibility of a synthetic component.

## Discussion

Marijuana is one of the most widely abused substances in the US. The effects of tetrahydrocannabinol (THC), the active ingredient in marijuana, have been well studied and reported substantially in the literature. When smoked, THC results in a rapid, dose-dependent tachycardia by 20-100%, an increase in blood pressure, and an increase in cardiac output by > 30%, leading to increased oxygen demand, which is augmented by the vasoconstriction from endothelial damage by smoking and activation of CB1 receptors by marijuana [3,4]. Anxiety, panic, impaired attention, and psychosis are commonly experienced as well. Several EKG abnormalities have been identified, including sinus tachycardia, premature ventricular contractions, left or right bundle branch blocks, and ventricular tachycardia or fibrillation [5,6].

Cardiovascular effects of marijuana include cardiac arrhythmias, increased risk of cardiomyopathy, sudden cardiac death, coronary artery dissection, and MI, which is a rare complication [7]. Proposed mechanisms are linked to the five-fold increase in carboxyhemoglobin, an increase in factor VII activity, and THC-induced hemodynamic stress, resulting in a lower angina threshold [8,9]. Such factors are implicated in contributing to the disruption of preexisting atherosclerotic plaque, resulting in coronary artery occlusion and subsequent MI, which has been reported in numerous cases in the literature. Mittleman et al. found the relative risk of MI to be 4.8 one hour after THC exposure and 1.7 after 2 hours [10]. Less commonly, the symptoms of MI are found to occur in patients exposed to marijuana in whom subsequent workup reveals patent and clean coronary arteries [11,12].

Synthetic cannabinoids, such as Spice and K2, are rapidly gaining popularity among recreational drug users. Despite being marketed as a natural, safer alternative to conventional cannabinoids, the active ingredient, JWH-018, has been found to be four- to five-fold as potent as THC and is associated with similar physiological effects [2,8]. Due to its chemical structure and weak monoamine oxidase (MAO) inhibition at high doses, serotonin syndrome is a potential concern. Perhaps most concerning to clinicians is that these synthetic cannabinoids are undetectable on standard UDS. It is critical to note, however, that it is common for synthetic and standard cannabinoids to be smoked as a mixture, and that if standard cannabinoids are found on UDS, the potential for synthetic cannabinoids ingestion must still be considered.

MI as a sequela of synthetic cannabinoid use is rare but has previously been reported both in the presence and absence of atherosclerotic coronary plaque [1,6,8]. Most commonly, this was reported among adolescent patients, the youngest reported patient being 16 years old by Toce et al [13]. Our patient was unusual in that his MI symptoms were due to global vasospasm of the coronary arteries as compared to localized spasm in the majority of cases reported, which we theorize was contributed to by marijuana toxicity, perhaps augmented by synthetic cannabinoids [4]. Synthetic cannabinoids are not routinely tested for. Mir et al. noted that in one patient who actively tested for synthetic cannabinoids, the result was negative despite the patient affirming to use of K2 [6]. It is important to note that hundreds of chemical variants are likely in circulation relative to the few that clinicians are capable of testing for. Moreover, as with all other synthetic substances, manufacturers of synthetic drugs are often aware of what may and may not be detected on screening and are accordingly able to stay ahead in the arms race.

The workup for such patients, therefore, is identical to the workup for any patient suspected of acute coronary syndrome (ACS). Patients presenting with symptoms of MI, even when substance use is suspected or known, must still be evaluated according to standard protocols based on risk stratification. Several of these patients will require ischemic workup, which may or may not reveal evidence of obstructed coronary vasculature. The diagnosis of substance-induced angina is a diagnosis of exclusion, with the particular substance identified through careful patient history, UDS, and the clinician’s awareness of current commonly abused substances.

## Conclusions

In patients with low to absent risk for cardiovascular events, particularly pediatric and young adult patients, presenting with symptoms of MI/cardiac arrest, substance-induced (marijuana) MI should be suspected. The detection of synthetic cannabinoids is not part of routine UDS. Therefore, the clinician must maintain a high index of suspicion when evaluating the patient and possible etiologies for their ACS. As it becomes legal for recreational purposes in the US, more literature needs to be published to raise awareness in the general population.
